# Inhibition of PANX1 Channels Reduces the Malignant Properties of Human High-Risk Neuroblastoma

**DOI:** 10.7150/jca.79552

**Published:** 2023-03-13

**Authors:** Stéphanie Langlois, Marie-Eve St-Pierre, Stephen H. Holland, Xiao Xiang, Emily Freeman, Hisham Mohamed, Ahmet Cem Dural, Ahmed Hammad, Sanaz Karami, Chloé van de Panne, Kyle N. Cowan

**Affiliations:** 1 Molecular Biomedicine Program, Children's Hospital of Eastern Ontario Research Institute, Ottawa, Ontario, Canada; 2 Department of Cellular and Molecular Medicine, University of Ottawa, Ottawa, Ontario, Canada; 3 Department of Surgery, Children's Hospital of Eastern Ontario, University of Ottawa, Ottawa, Ontario, Canada

**Keywords:** Cancer, Carbenoxolone, Neuroblastoma, Pannexin, PANX1, Probenecid, Xenografts

## Abstract

Pannexin 1 (PANX1) is expressed in many tissue types including tissues of neural origin. Neuroblastoma (NB) is a neural crest-derived malignancy mainly occurring in children. The majority of NB patients present with high-risk disease for which current therapies are ineffective. Here, we show that while PANX1 is expressed in NB of all stages, high *PANX1* expression in high-risk NB is associated with a reduced survival probability. PANX1 channel inhibition using probenecid (PBN) or carbenoxolone (CBX) reduced the proliferation of our panel of high-risk NB cell lines. We show that expression of the Y10F PANX1 mutant, which cannot be phosphorylated on tyrosine 10 and acts in a dominant-negative manner, curtailed NB cell proliferation. Furthermore, PBN and CBX treatment halted the growth of NB spheroids and in some cases triggered the regression of established NB spheroids. Finally, both drugs reduced the progression of high-risk NB *in vivo*. Together our data indicate that PANX1 channels regulate human NB malignant properties and that the use of PBN or CBX may provide a new therapeutic approach for high-risk NB.

## Introduction

Neuroblastoma (NB) is the most common extracranial solid tumor in childhood, accounting for 7-10% of pediatric cancers and 15% of all pediatric cancer deaths 1, 2. It is widely accepted that the cell origin for NB arises from the sympathoadrenal lineage of the neural crest during development 3. The neural crest is present during embryogenesis and gives rise to diverse cell types including enteric neurons and glia, as well as peripheral neurons 3. At diagnosis, most NB are located in the abdomen, associated with the adrenal gland or sympathetic ganglia 3. NB are classified into risk groups based on the International Neuroblastoma Risk Group (INRG) classification system. Various factors are used to stratify patient risk such as: 1) age at diagnosis; 2) stage defining the extent of the disease by the International Neuroblastoma Staging System (INSS) (stage 1, 2A, 2B, 3, 4, or 4S); 3) tumor histology using the International Neuroblastoma Pathology Classification criteria; 4) *MYCN* status; and 5) DNA index or tumor cell ploidy 4, 5. Based on this, clinically different pretreatment groups are defined as very low, low, intermediate, and high-risk in terms of their associated 5-year event-free survival 2. While patients with low and intermediate risk disease have an excellent prognosis, most patients present with metastatic high-risk disease. Despite current treatment regimens that include multimodal cytotoxic therapies and aggressive surgery, the survival rate of high-risk NB patients remains below 50%, while patients with refractory or relapsed tumors have no cure 6. Altogether, this highlights the urgent need to improve high-risk NB treatment, decrease relapse, and prevent disease progression.

Pannexin 1 (PANX1 in human; Panx1 in rodents), which forms homo-heptameric single membrane channels permeable to ion and metabolite 7, 8, is expressed in many tissue types including tissues of neural origin 9. Pannexin 1 individual subunits consist of four α-helical transmembrane (TM) domains with one intracellular loop between TM2 and TM3, as well as intracellular N- and C-terminal domains 8. PANX1/Panx1 expression is tightly regulated in the context of differentiation and development in various cell and tissue types 10-15 and are altered in a variety of diseases including cancer 11, 16-19. Moreover, once translated, PANX1/Panx1 is subjected to post-translational modifications (PTMs), which subsequently affect channel trafficking and function 20. Specifically, PANX1/Panx1 is known to be subjected to N-linked glycosylation on its second extracellular loop 8, 21, 22, which has been suggested to increase its trafficking to the cell surface 8, 23. S-nitrosylation of the cysteine residue 40 (N-terminus) or 346 (C-terminus) of PANX1 inhibits its function 24, while caspase cleavage of PANX1 at is C-terminus irreversibly activates channel opening 25, 26. PANX1 function can also be regulated by phosphorylation at its tyrosine (Y) residues Y150 (first intracellular loop), Y198 (intracellular loop), and Y308 (C-terminus) 27-30. Interestingly, a phosphoproteomic study screening of human NB cell lines, in which 1662 phosphorylated proteins were detected, unveiled a novel PANX1 phosphorylation site at Y10 (N-terminus) 31. The PANX1 phosphopeptide containing Y10 (TEY*VFSDFLLK) was identified in the non-soluble, soluble, and endosomal fractions of the SK-N-Be(2) cell line expressing TrkA 31. This phosphorylation site has also been curated in the online database PhosphoSitePlus from two separate mass spectrometry data depositions, one from the human SK-N-Be(2) cell line and the other one from mouse brain tissue (https://www.phosphosite.org/siteGroupAction.action?id=23129120&protOrg=14530&showAllSites=true&showHTPRefsOnly=true), but the role of this post-translational modification remained to be investigated.

Numerous studies have now shown a role of human PANX1 in cancer including breast cancer 17, 32, gastric cancer 33, hepatocellular carcinoma 34, melanoma 35, and rhabdomyosarcoma 18, 36, 37. Notably, the murine NB-derived cell line N2a was shown to express Panx1, which positively mediated proliferation of these cells through release of ATP 9. As such, probenecid (PBN), a pannexin 1 channel blocker 38, significantly reduced N2a cell proliferation 9. In addition to PBN, carbenoxolone (CBX) is also routinely used to block PANX1/Panx1 channel activity *in vitro* and *in vivo* 32, 38-41 by interacting with its first extracellular loop 42. PBN and CBX have already been used in clinic for other applications 43, 44, thus providing an interesting drug repurposing possibility. Moreover, it has been demonstrated that inhibition of PANX1 channels by PBN and CBX treatment decreased cell growth and migration of human melanoma cell lines *in vitro*, as well as melanoma tumor weight and invasiveness in chicken embryo xenografts 35. Since NB and melanoma share a common origin as neural crest-derived tumors 45, treatment with PBN and CBX may also be beneficial for high-risk NB.

Here we show that PANX1 is expressed in NB tumors of all stages. However, high *PANX1* expression is associated with a reduced overall and event-free survival probability in high-risk patients, while low expression predicted a better prognosis. Our panel of seven patient-derived high-risk NB cell lines express PANX1 channels. Using these cell lines, we found that treatment with PBN or CBX consistently reduced high-risk NB cell proliferation *in vitro*. Furthermore, treatment with PBN or CBX abolished NB 3D spheroid formation and induced regression of established NB spheroids. We further show that a PANX1 mutant (Y10F) resistant to phosphorylation at tyrosine residue 10 has decreased channel activity and acts in a dominant-negative manner. When expressed in NB cell lines, the Y10F PANX1 mutant slowed their proliferation; further strengthening the concept that PANX1 channels promote NB malignancy and that their inhibition reduces NB malignant properties. *In vivo*, treatment with PBN or CBX significantly curtailed the growth of high-risk NB xenografts in mice. Collectively, this work suggests that PANX1 may be a promising new therapeutic target for NB and reveals the potential benefit of using PBN and CBX for high-risk NB treatment.

## Materials and Methods

### Tumor Specimens and Cell Lines

**Figure 1:**
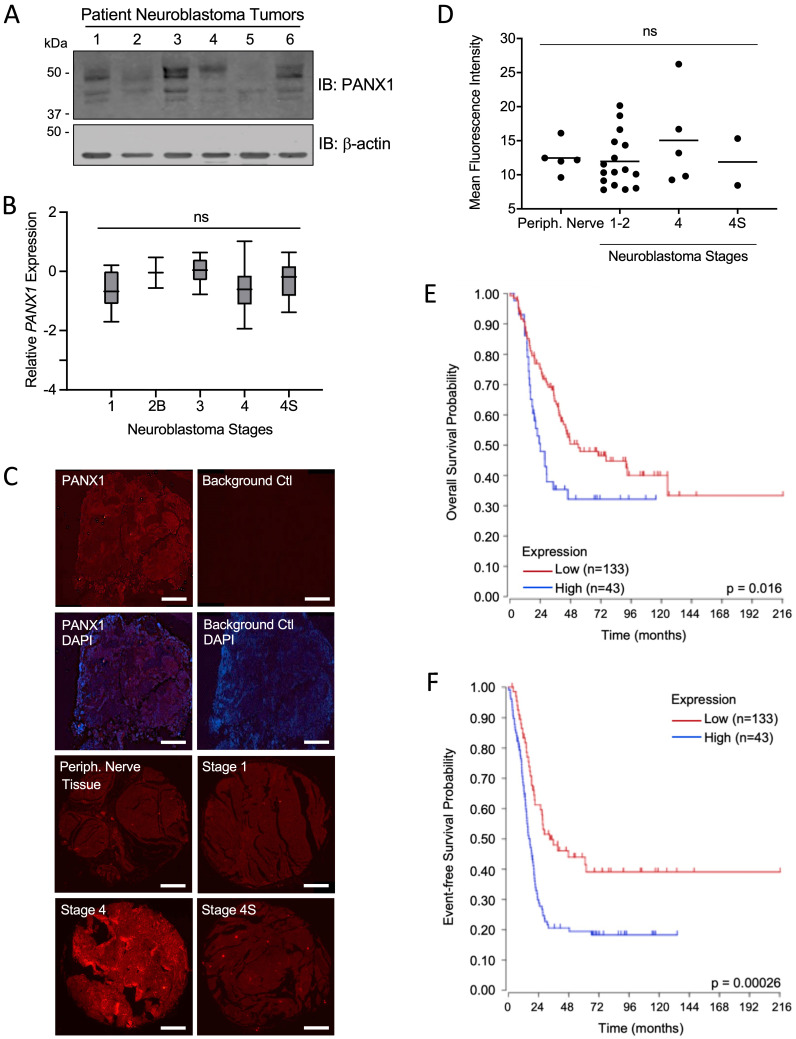
** PANX1 is expressed in all NB stages, but high *PANX1* expression is associated with a reduced survival probability in high-risk NB patients. (A)** Representative Western blot showing PANX1 levels (50 µg per well) in 6 pediatric NB tumor specimens (clinical information in Materials and Methods in 'Tumor Specimens and Cell Lines'). ß-actin was used as a loading control (n=2 technical replicates). **(B)** *PANX1* expression across various stages of neuroblastoma as classified by the International Neuroblastoma Staging System (INSS) (ns: not statistically significant; one-way ANOVA followed by Tukey's *post hoc* tests). **(C)** Representative pictures of PANX1 (red) staining in a NB specimen compared to that of the negative control (Background Ctl) performed in the absence of primary (PANX1) antibodies (top row right). The second row includes DAPI (blue) staining to help delineate the tumor area. The third and fourth rows show representative pictures of PANX1 staining (red) in normal peripheral nerve tissue (Periph. Nerve Tissue), as well as in stage 1, stage 4, and stage 4S NB present in the tissue array. Bar = 250 μm. **(D)** Quantification (Mean Fluorescent Intensity) of PANX1 staining in normal peripheral nerve tissue (Periph. Nerve; n=5), stage 1-2 (n=16), stage 4 (n=5), and stage 4S (n=2) NB specimens present in the tissue array (ns: not statistically significant; one-way ANOVA followed by Tukey's *post hoc* tests). **(E)** Kaplan-Meier analysis showing the overall survival probability of high-risk NB patients with high (n=43) or low (n=133) *PANX1* expression (*p* = 0.016; Log-rank test).** (F)** Kaplan-Meier analysis showing the event-free survival probability of high-risk NB patients with high (n=43) or low (n=133) *PANX1* expression (*p* = 0.00026; Log-rank test).

After institutional ethics board approval (CHEO Research Ethics Board, protocol 15/46X), six archived frozen NB tumor samples were obtained, as secondary use of clinical samples, from the Department of Pathology and Laboratory Medicine, Children's Hospital of Eastern Ontario (CHEO), Ottawa, Ontario, Canada. These primary tumor samples were from pediatric patients that had a diagnosis of NB, had undergone surgical resection, and for which excess tissue was not needed for clinical purposes. When such archived excess tissue was preserved in a way that is suitable for Western blotting (frozen without fixation) and thus available for our use, they were included in the current study. The clinical parameters of the patients from which the tumor samples (#1-6 as labelled in Figure 1A) were obtained are: **#1**: male, 2 months old, left adrenal gland NB tumor, stroma-poor, poorly differentiated with low MKI, favorable histology, MYCN non-amplified, stage 4S; **#2**: female, 5 months old, left suprarenal tumor, Schwannian stroma-poor, poorly differentiated with intermediate MKI, favorable histology, MYCN non-amplified, stage 4; **#3**: female, 3 years and 7 months old, retroperitoneal mass, Schwannian stroma-poor NB, poorly differentiated with low MKI, unfavorable histology, MYCN non-amplified, stage 2B; **#4**: male, 3 years and 10 months old, right adrenal mass, Schwannian stroma-rich, differentiated favorable histology, MYCN non-amplified, stage 4; **#5**: male, 1 month old, neck NB tumor, Schwannian stroma-poor, poorly differentiated with low MKI, MYCN non-amplified, stage 1; and **#6**: female, 22 months old, left adrenal NB tumor, stroma-poor, undifferentiated/poorly differentiated with low MKI, unfavorable histology, stage 1. Experiments were carried out in accordance with the ethical principles set for in the Declaration of Helsinki and with the CHEO Ethics Board guidelines and regulations.

The GI-L-IN, GI-M-EN, IMR-32, Lan-1, SK-N-AS, SH-SY5Y, and SK-N-Be(2) NB cell lines were a gift from Dr. R. Korneluk (Children's Hospital of Eastern Ontario Research Institute, Ottawa, ON, Canada). The HEK293T cell line were purchased from the American Type Culture Collection. Ad293 cells, which are derivative of human embryonic 293 cells with improved cell adherence, where a kind gift from Dr. D.W. Laird (Western University, London, ON, Canada). The Rh30 cell line was a gift from Dr. P. Houghton (St. Jude Children's Hospital, Memphis, TN, USA). All cell lines were grown in DMEM (Dulbecco's modified Eagle's medium) supplemented with 10% fetal bovine serum (FBS), 1% penicillin, 1% streptomycin, and 1% L-glutamine at 37^0^C in 5% CO_2_.

### Bioinformatic Analysis of PANX1 Expression Neuroblastoma

The microarray data for comparing *PANX1* expression across pediatric neuroblastoma stages were retrieved from USCS Xena Brower (https://xenabrowser.net/) under the “Neuroblastoma (Khan)” study group using “*PANX1* gene expression” as the first variable and “stage” as the second variable 46. This dataset consisted of 7 stage 1, 2 stage 2B, 11 stage 3, 123 stage 4, and 16 stage 4S NB tumor specimens, which was generated by Krasnoselsky et al. 47 and deposited into Oncogenomics DB (https://omics-oncogenomics.ccr.cancer.gov/cgi-bin/JK). The retrieved *PANX1* expression data were regrouped per stage according to INSS, irrespective of the *MYCN* status. The R2 platform was used to analyze high-risk NB patient data sets (n=176) from the SEQC cohort (SEQC-498-seqcnb1 dataset). Based on the median value of *PANX1* mRNA in the tumors, high-risk NB patients were classified into low and high *PANX1* expression groups. Kaplan-Meier curves for overall survival probability and event-free survival probability were generated and *p* values obtained using the R2 platform.

### Tissue Array and PANX1 Immunolabeling

Neuroblastoma and peripheral nerve tissue array (MC602; US Biomax, Rockville, MD) was deparaffinized in xylene (2 washes, 5 minutes each) and rehydrated in graded alcohols (100% ethanol, 2 minutes; 95% ethanol, 2 minutes; 70% ethanol, 2 minutes; followed by two washes in deionized water, 5 minutes each). Antigen retrieval was performed using Tris-EDTA pH 9.0 at 95^o^C for 20 minutes. Following blocking in 2% bovine serum albumin (BSA) for one hour at room temperature, the tissue array was labeled with anti-PANX1 (Sigma-Aldrich, Cat#: HPA016930, 1:50) for one hour at 37 ºC and then washed 3 times in PBS, 10 minutes per wash at room temperature. After the washes, the tissue array was incubated with anti-rabbit secondary antibodies conjugated to Alexa Fluor 594 (Thermo Fisher Scientific, Waltham, MA, Cat#: A-11012, 1:200) for one hour at 37 ºC, followed by 3 washes with and a final wash in deionized water for 15 minutes. The background control was performed without incubating with anti-PANX1 antibodies. The slides were mounted using Fluoromount-G medium with DAPI (Invitrogen, Cat#: 00-4959-52) and visualized with the slide scanner Zeiss Axio Scan.Z1 (Toronto, ON, Canada). Quantification (mean fluorescence intensity) was done using ImageJ and averaged from three random field of the same area for each tumor core.

### Transfection and Stable Cell Lines Generation

Cells were transfected using Lipofectamine 2000 (Thermo Fisher Scientific) reagent according to manufacturer's protocol. SK-N-Be(2) cells were transfected with 10 nM of Silencer Select Pre-designed siRNA targeting *PANX1* (sense: CGAUCAGUUUCAGUGCAAAtt; anti-sense: UUUGCACUGAAACUGAUCGgg) or its control non-targeting sequence (Silencer Select Negative Control No. 1 siRNA, Cat#: 4390843) and analyzed by Western blotting 96 hours post-transfection. HEK293T cells were transfected with myc-PANX1, myc-Y10F, or E.V. (empty vector: pCDH-CuO-MCS-EF1-GFP) and submitted to Western blot analysis or deglycosylation assay (described below in the 'Western Blotting' and 'Deglycosylation Assay' subsections) 48 hours later. Ad293 cells were co-transfected with 1:1 ratio of myc-PANX1 + E.V., myc-Y10F + E.V., myc-PANX1 + myc-Y10F, or E.V. alone and submitted to dye uptake assay (described below in the 'Dye Uptake Assay' subsection) 48 hours later. SK-N-AS transfected with myc-Y10F or the empty vector (E.V.) were also submitted to dye uptake assay (described below in the 'Dye Uptake Assay' subsection) 48 hours post-transfection. LAN-1, SK-N-AS, SK-N-Be(2), and SH-SY5Y cells were transfected with myc-PANX1, myc-Y10F or the empty vector control and submitted to the BrdU proliferation assay (described below in the 'Proliferation Assay' subsection) 48 hours later.

SK-N-Be(2) cell line expressing a non-silencing shRNA control (Ctl shRNA, Cat#: RHS4743) or *PANX1* shRNA (Cat# RHS4740-NM-015368) were generated by transduction using Inducible Dharmacon TRIPZ Lentiviral shRNAs (Horizon Discovery, Cambridge, UK). Lentiviral particles were produced using the Trans-Lentiviral shRNA Packaging System (Cat#: TLP5912, Horizon Discovery) in HEK293T cells. Selection was done using 4 µg/ml puromycin and cells were treated for 96 hours with 2 µg/ml doxycycline to induce shRNA expression prior to analysis of PANX1 levels. The NB stable cell lines expressing GFP (SK-N-AS, SH-SY5Y, GI-LIN, and SK-N-Be(2)) used for the spheroid assay were generated by transduction with the pCDH-CuO-MCS-EF1-GFP lentivector (System Biosciences, Palo Alto, CA, Cat#: QM513B-1) packaged into lentiviral particles using the pPACKH1 Lentivector Packaging Kit (System Biosciences, Cat#: LV500A-1). Transduced cells were selected using 4 µg/ml puromycin.

The CRISPR knockout Rh30 cell lines were generated as previously described 48 using two independent guide pairs from the transEDIT-dual CRISPR Whole Genome Arrayed Library (Transomic Technologies, Huntsville, AL) targeting *PANX1* with sequences 5'-GATGGTCACGTGCATTGCGG and CCTCTACAATCTCTTCTTGG in pCLIPdual (KO-1); or 5'-GCAGAGATGAGAGAGGAGCA and 5'-AATCGAGATCTCCTGCGCGA (KO-2) independently cloned into pSpCas9(BB)-2A-Puro 48. Plasmids carrying these guide pairs were transfected independently into Rh30 cells with a 1:1 mass ratio of pLentiCas9Blast 49. Transduced cells were selected using 5 µg/ml puromycin. T7 endonuclease assays were carried out using T7 endonuclease I (NEB) according to the manufacturer's recommendations on PCR products generated using the following primer pairs: 5'-GGTACTTGGTTTCCCCGCAT and 5'-GGGATGCGAGCAGAGTAGAC; or 5'-CAAGTACATTAGCTGCCGCC and 5'-CTGCAAAGGCTCTCGGTAGA. These *PANX1* knockout Rh30 cell lines were generated by the Genome Engineering and Molecular Biology Core at the University of Ottawa.

### Generation of the myc-Y10F and myc-N255A PANX1 Mutants

Myc-PANX1, tagged to its C-terminus, was obtained from OriGene (Origene, Rockville, MD, Cat#: RC204474), subsequently PCR-amplified using sequence-specific primers (fwd: 5' TCACAATCTAGAGCCGCCGCGATCGCCATGGCCATCGCTCAC 3'; rev: 5' GTCGCAGAATTCCTACTTATCGTCGTCATCCTTGTAATC 3') and cloned into the XbaI and EcoRI restriction sites of a Lentivirus dual promoter expression vector pCDH-CuO-MCS-EF1-GFP obtained from System Biosciences (Cat#: QM513B-1). After verification by Sanger sequencing, the pCDH-CuO-Myc-PANX1-EF1-GFP construct (now referred here as myc-PANX1) 36 was used as a template to generate the myc-tagged Y10F PANX1 (myc-Y10F) and the myc-tagged N255A (myc-N255A) mutants. To this end, the QuickChange site-directed mutagenesis kit (Agilent Technologies, Santa Clara, CA, USA) was used with the following primers: Y10F fwd: CCGAGAACACGAACTCCGTGGCCAGGTG; Y10F rev: CACCTGGCCACGGAGTTCGTGTTCTCGG; N255A fwd: GCATCAAATCAGGGATCCTGAGAGCCGACAGCACCGT; and N255A rev: ACGGTGCTGTCGGCTCTCAGGATCCCTGATTTGATGC. The myc-Y10F PANX1 mutant (tagged to its C-terminus) encodes a change from tyrosine (Y) to phenylalanine (F) at position 10, and the myc-N255A PANX1 mutant (tagged to its C-terminus) encodes a change from asparagine (N) to alanine (A) at position 255. Constructs were verified by sequencing.

### RNA Extraction, Reverse Transcription, and Quantitative PCR Analysis

Total RNA was extracted using RNeasy Mini Kit (Qiagen, Valencia, CA) and the reverse transcription was performed using High-Capacity cDNA Reverse Transcription Kit (Thermo Fisher Scientific) per the manufacturers' protocols. The synthesized cDNA was used as the template for quantitative PCR using iQ™ SYBR^®^ Green Supermix kit (BioRad, Hercules, CA) on Mastercycler ep *realplex* (Eppendorf, Mississaugua, ON, Canada) real-time PCR system with validated All-in-One qPCR primers for human *PANX1* (Cat#: HQP006368; fwd: GCTGTTCAGCAGAAGAACTCAC, rev: TCTGAGCAAATATGAGGAGCAG) and *β-actin* (Cat#: HQP016381; fwd: CCAACCGCGAGAAGATGA; rev: CCAGAGGCGTACAGGGATAG) (GeneCopoeia, Rockville, MD). Relative expression levels were determined using the comparative Ct method. Results were normalized to Human Neuron Total RNA (prepared from early passage neurons from human brain, Cat#: 1525, ScienCell, Carlsbad, CA).

### Western Blotting

 Tumor samples were homogenized in 1% SDS containing protease and phosphatase inhibitors (protease inhibitor cocktail (Cat#: 4693159001, Roche) and phosSTOP (Cat#: 04906837001, Roche) tablets) using the Omni Bead Ruptor (Omni International, Kennesaw, GA) with 2.38 mm metal beads. Cells were lysed on ice for 30 minutes using 1% Triton X-100, 150 mM NaCl, 10 mM Tris, 1mM EDTA, 1 mM EGTA. 0.5% NP-40, protease inhibitor cocktail (Cat#: 4693159001, Roche) and phosSTOP (Cat#: 04906837001, Roche) tablets (pH 7.4). Cell lysates (supernatants) were collected following a centrifugation at 12000 rpm for 10 minutes at 4 ºC. Protein concentrations were determined using the Pierce BCA Protein Assay Kit (Thermo Scientific, Cat#: 23227). Human Neuron Lysate (prepared from early passage neurons from human brain, ScienCell, Carlsbad, CA, Cat#: 1526) was used for comparison of PANX1 levels. Equal amounts of proteins (indicated in the respective panels of the figure legends) were separated by SDS-PAGE, transferred to PVDF membranes and blocked for one hour at room temperature using the Intercept blocking buffer (LI-COR Biosciences, Lincoln, NE). The membranes were incubated with antibodies against PANX1 (Sigma-Aldrich, Cat#: HPA016930, 1:1000), myc (R&D Systems, Minneapolis, MN, Cat #: #2276S, 1:1000), myc (Cell Signaling, Cat#: 2278S, 1:1000), tubulin (Santa Cruz, Cat#: sc-8035, 1:1000), β-actin (C4) (Santa Cruz, Cat#: sc-47778, 1:1000), or GAPDH (Advanced ImmunoChemical, Long Beach, CA, Cat#: 2RGM2, 1:1000). Membranes were washed three times (0.1% Tween 20 in PBS; 15 minutes per wash) and then incubated with goat anti-rabbit Alexa Fluor 680 (1:5000, Thermo Fisher Scientific, Cat#: A21109) or IRDye 800CW goat anti-mouse (1:5000, LI-COR Biosciences, Cat#: 925-32210) secondary antibodies for one hour at room temperature. Following three washes (0.1% Tween 20 in PBS; 15 minutes per wash), immunoblots were processed using the Odyssey infrared imaging system (LI-COR Biosciences).

### Deglycosylation Assay

 Deglycosylation assays were performed as described previously 10, 19. Briefly, equal amounts of cell lysates (100 µg) were subjected to deglycosylation treatment, with or without PNGase F (Protein Deglycosylation Mix II, Cat#: P6044, New England Biolabs), following manufacturer's instructions. To this end, lysates were first incubated with the Deglycosylation Mix Buffer 2 for 10 minutes at 75 ºC and allowed to cool down. Samples were then incubated with the Protein Deglycosylation Mix II at room temperature for 30 minutes, and then at 37 ºC for one hour. Finally, samples were analyzed by Western blotting with anti-PANX1 or anti-myc antibodies, as described in the Western blotting subsection.

### Dye Uptake Assay

Transfected Ad293 cells were exposed for 30 minutes at 37 ºC to control (low K^+^: 145 mM NaCl, 5 mM KCl, 1.4 mM CaCl_2_, 1.0 mM MgCl_2_, 10 mM HEPES, pH 7.4) and high K^+^ (60 mM NaCl, 50 mM KCl, 1.4 mM CaCl_2_, 1.0 MgCl_2_, 10 mM HEPES, pH 7.4) solutions containing 2 mg/ml sulforhodamine B dye with or without PBN (1 mM, Molecular Probes, Cat#: P36400) or CBX (100 µM, Sigma, Cat#: C4790) 50. The cells were washed 10 times prior to examining the dye uptake under a fluorescent microscope (EVOS FL Auto Imaging System). Ten fluorescent and their corresponding phase-contrast image fields were collected using a 20x objective. Cells were then harvested, and lysates submitted to Western blot analysis using anti-PANX1 and anti-GAPDH. The % of dye uptake incidence was calculated as ((the number of cells that took the dye/the total number of cells) x 100) for each of the ten field, averaged for each condition, and normalized to PANX1 levels.

Sulforhodamine B dye uptake assay (2 mg/ml in Dulbecco's-PBS (1x PBS plus 0.1 g/l CaCl_2_ anhydrous and 0.1 g/l MgCl_2_.6H_2_O), pH 7.4)) following mechanical stimulation was performed in NB cell lines in the absence or presence of PBN 22. To this end, the cells were pre-treated with 1 mM PBN (or saline for the vehicle control) for 30 min at 37 ºC. The cells were then washed twice with Dulbecco's-PBS on ice and stimulated with a continuous drip of 800 µl of sulforhodamine B dye solution (with or without 1 mM PBN) from a height of 2.5 cm from the cells. The stimulation was repeated twice, and the cells were then incubated with the dye for 5 minutes on ice. The cells were washed 10 times with cold Dulbecco's-PBS prior to examining the dye uptake under a fluorescent microscope (Olympus IX51). Four fluorescent and their corresponding phase-contrast image fields were collected at the drip target site using a 20x objective. The % of dye uptake incidence was calculated as ((the number of cells that took the dye/the total number of cells) x 100) for each of the four fields and then averaged for each experiment. Transfected SK-N-AS cells were submitted to dye uptake following the same mechanical stimulation protocol.

### Proliferation Assay

For the proliferation assay with the NB cell lines treated with PBN or CBX, a colorimetric BrdU Cell Proliferation ELISA Kit (Cat#: 11647229001, Roche, Laval, QC, Canada) was used (96-well plate). Briefly, cells (1x10^4^ cells/well for PBN treatment; 1x10^5^ cells/well for CBX treatment) were treated with 1 mM PBN (Molecular Probes, Cat#: P36400), 100 µM CBX (Sigma, Cat#: C4790), or the saline vehicle control for a total of 48h, refreshing the inhibitors after 24h. BrdU labeling was done 2 hours before the end of the incubation period after which the cell media was aspirated. Fixation/denaturation, incubation with anti-BrdU antibody, washing, and substrate reaction steps were performed as per the manufacturer's protocol. The absorbance was measured in a microplate reader (Synergy HTX plate reader, BioTek, VT) at 370 nm.

For the over-expression experiments, cells were transfected with the empty vector (E.V.), myc-PANX1, or myc-Y10F. Forty-eight hours post-transfection, cells were incubated with 10 µM BrdU Labeling Reagent (Invitrogen, Cat#: 000103) at 37 ºC. Based on their different growth rates, SK-N-Be(2) cells were incubated with BrdU for 2 hours, while LAN-1 cells were incubated for 4 hours, and SK-N-As and SH-SY5Y cells were incubated for 6 hours. Briefly, following fixation the cells were blocked with 2% BSA with 0.1% Triton X-100 for 45 minutes, and then incubated in 2N HCl for 20 minutes to denature the nuclear DNA. The cells were labeled sequentially with anti-BrdU (1:200; Thermo Fisher Scientific, Cat#: 03-3900), Alexa Fluor 594 conjugated anti-mouse IgG (1:500, Thermo Fisher, Cat#: A-11005), anti-myc (1:200; Cell Signaling, Cat#: 2276S) and Alexa Fluor 488 conjugated anti-rabbit IgG (1:500, Thermo Fisher Scientific, Cat#: A-11008) with PBS washes in between. Incubations with antibodies were one hour each at room temperature. The labeled cells were then mounted using Fluoromount-G medium with DAPI (Invitrogen, Cat# 00-4959-52). Ten random field images per sample were taken using a fluorescent microscope equipped with a 20X objective and the number of BrdU positive nuclei from total transfected cells were counted.

### Spheroid Formation and Regression Assays

3D spheroid assay was performed and quantified using Incucyte Live Cell Imaging System (Essen BioScience Inc., Ann Arbor, MI) and its accompanying software. Cells (SK-N-AS: 20000 cells/well; SH-SY5Y: 30000 cells/well; GI-L-IN: 20000 cells/well; SK-N-Be(2): 3000 cells/well) were seeded in Ultra Low Adhesion (ULA) 96-well plates (Corning, Kennebunk, ME). Plates were centrifuged at 1000 rpm for 10 minutes at room temperature prior to be placed at 37 ºC in the Incucyte incubator. In the spheroid formation experiment, cells were treated at the time of seeding and then daily with 1 mM PBN, 100 µM CBX, or the saline vehicle control. In the spheroid regression experiment, treatment started only once the spheroids had formed (after 48-72 hours of seeding) and then daily afterwards. Mean image fluorescence, a surrogate measurement of 3D tumor growth, was quantified using Incucyte Live Cell Imaging System every 2 hours for 190-320 hours 18.

### In Vivo Tumor Xenograft Growth

All experiments were approved by the Animal Care Committee at the University of Ottawa and conducted per the guidelines of the Canadian Council of Animal Care (protocol CHEOe-2680). Twenty-seven million SK-N-Be(2) cells (13 mice for saline; 9 mice for PBN; 7 mice for CBX treatment) and one million SH-SY5Y cells (8 mice for saline; 6 mice for PBN; 7 mice for CBX treatment) resuspended in 1:1 DMEM:Matrigel (Matrigel Matrix High Concentration (HC) Growth Factor Reduced (GFR), Corning, Cat#: 354263) were injected subcutaneously in the flank of 4-week-old female CD1 nu/nu mice (Charles River Laboratories, Wilmington, MA). Mice were randomly assigned prior to treatment. Mice were treated every day by intraperitoneal injection with PBN (250 mg/kg body weight; Sigma, Cat#: P8761) 51, 52, CBX (30 mg/kg; Sigma, Cat#: C4790) 53, or saline. Tumors were measured every 2-3 days using a digital caliper. Mice were sacrificed when tumors reached ~2000 mm^3^ (V = L x W^2^ / 2 (L= length; W=width of tumor)) as per our institutional guidelines. Injections and tumor measurement were performed in a double blinded matter.

### Statistics

Unpaired or paired two-tailed student's *t*-tests, one-way ANOVA followed by Tukey's *post hoc* tests, Log-rank or the Gehan-Breslow-Wilcoxon tests were used as indicated in the figure legends. Results with *p* < 0.05 were considered significant (n ≥ 3 independent biological replicates). Statistical analysis was performed using the GraphPad Prism software.

## Results

### PANX1 is expressed in all NB stages, but high PANX1 expression is associated with a reduced survival probability in high-risk NB patients

First, we examined PANX1 expression in six NB tumors from pediatric patients and found that, while levels varied between specimens, PANX1 protein was detected in all tumors (Fig. 1A). Using *PANX1* microarray data from the 'Neuroblastoma (Khan)' study group 47, we found that there were no significant differences in *PANX1* mRNA expression between pediatric NB at various stages based on the INSS system (Fig. 1B). PANX1 was also immunolabeled in 25 NB tumors (stage 1, 2, 4, and 4S) and 5 normal peripheral nerve tissue (Periph. Nerve Tissue) samples in a tissue array slide and quantified. No labeling was detected in the absence of primary antibody (Background Ctl; Fig. 1C). PANX1 was expressed in peripheral nerve tissue and all NB tumors (Fig. 1C, D). Like *PANX1* mRNA, there were no significant difference in PANX1 protein levels between the various stages (Fig. 1D). We then used the R2 platform to analyze high-risk NB patient data sets (n=176) from the SEQC cohort (SEQC-498-seqcnb1 dataset). Based on the median value of *PANX1* mRNA in the tumors, high-risk NB patients were classified into low (n=133) and high (n=43) *PANX1* expression groups. As shown in Figure 1E, the Kaplan-Meier survival curves indicate that high *PANX1* expression may be correlated with a worse overall survival compared to high-risk NB patients with low *PANX1* expression (*p* = 0.016). High *PANX1* expression in high-risk NB patients is also associated with a significantly reduced event-free survival (Fig. 1F; *p* = 0.00026), suggesting a worse outcome for these patients.

### Patient-derived high-risk NB cell lines express PANX1 channels

**Figure 2:**
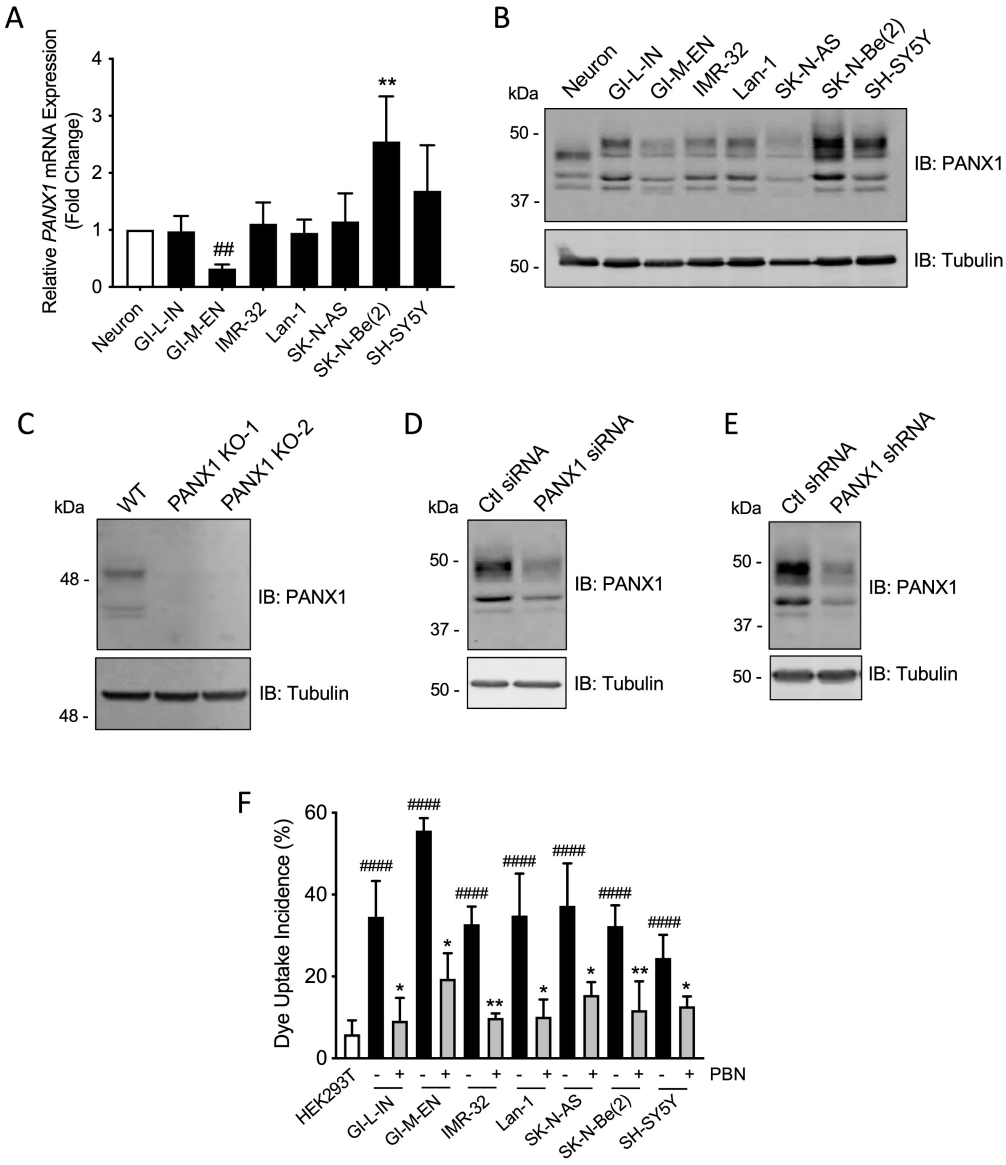
** Patient-derived high-risk NB cell lines express PANX1 channels.** PANX1 mRNA and protein levels in human neurons and in seven high-risk NB cell lines were assessed by **(A)** quantitative real-time PCR (n=4 independent biological replicates; ** *p*<0.01 compared to neurons and the other NB cell lines; ^##^ *p*<0.01 compared to SH-SY5Y (one-way ANOVA followed by Tukey's *post hoc* tests)) and **(B)** Representative Western blotting (n=3 independent biological replicates; 50 µg per well). Tubulin was used as a loading control for the Western blot. **(C)** PANX1 was detected in wild type (WT) Rh30 cells, but not in the *PANX1* knockout (KO-1 and KO-2) Rh30 cell lines (n=3 independent biological replicates; 30 µg per well). Tubulin was used as a loading control. PANX1 was detected in SK-N-Be(2) cells in which PANX1 was down-regulated using siRNA **(D)** or shRNA **(E)**, compared to the non-targeting control (Ctl) siRNA and shRNA sequences (n=3 independent biological replicates; 50 µg per well). Tubulin was used as a loading control. **(F)** Dye uptake incidence following mechanical stimulation was measured in the seven NB cell lines in the absence or presence of 1 mM PBN. Dye uptake was also measured in HEK293T cells, which are devoid of pannexins, as a comparison. ^####^* p*<0.0001 compared to HEK293T cells (unpaired two-tailed student' s *t*-tests); * *p*<0.05; ** p<0.01 compared to their counterparts treated with the vehicle control (n≥3 independent biological replicates; paired two-tailed student's *t*-tests).

To further examine PANX1 expression in high-risk NB, quantitative real-time PCR and Western blotting were performed on a panel of seven patient-derived high-risk NB cell lines compared to neurons. *PANX1* transcript levels were similar between most NB cell lines and neurons (Fig. 2A). However, the SK-N-Be(2) cell line displayed a significant increase in *PANX1* mRNA, while the GI-M-EN cell line expressed less *PANX1* mRNA when compared to SK-N-Be(2) and SH-SY5Y (Fig. 2A) cell lines. Accordingly, PANX1 protein was detected in all NB cell lines (Fig. 2B). Notably, a higher molecular weight PANX1 species (~50 kDa) was present in NB cell lines, but not in neurons, likely reflecting different post-translational modifications such as glycosylation and phosphorylation 20. To demonstrate the specificity of this antibody, the rhabdomyosarcoma cell line Rh30, which expresses PANX1 37, was used to generate knockout (KO) controls utilizing the CRISPR-Cas9 system with two independent guide pairs (KO-1 and KO-2). As shown in Figure 2C, PANX1 was detected in the wild-type Rh30 cell line, but not in the KO counterparts. In the SK-N-Be(2) cell line, the levels of all species were reduced using PANX1 siRNA (Fig. 2D) or shRNA (Fig. 2E) confirming that they correspond to PANX1. Next, all NB cell lines displayed a significant increase in sulforhodamine B dye uptake incidence following mechanical stimulation, compared to pannexin-devoid HEK293T cells that showed minimal dye uptake (Fig. 2F). Dye uptake incidence in all NB cell lines was significantly reduced in the presence of 1 mM PBN, indicating that this activity was due to PANX1 channels (Fig. 2F). All together, these results indicate that human high-risk NB cell lines express PANX1 channels.

### Treatment with PBN or CBX inhibits NB cell proliferation

**Figure 3:**
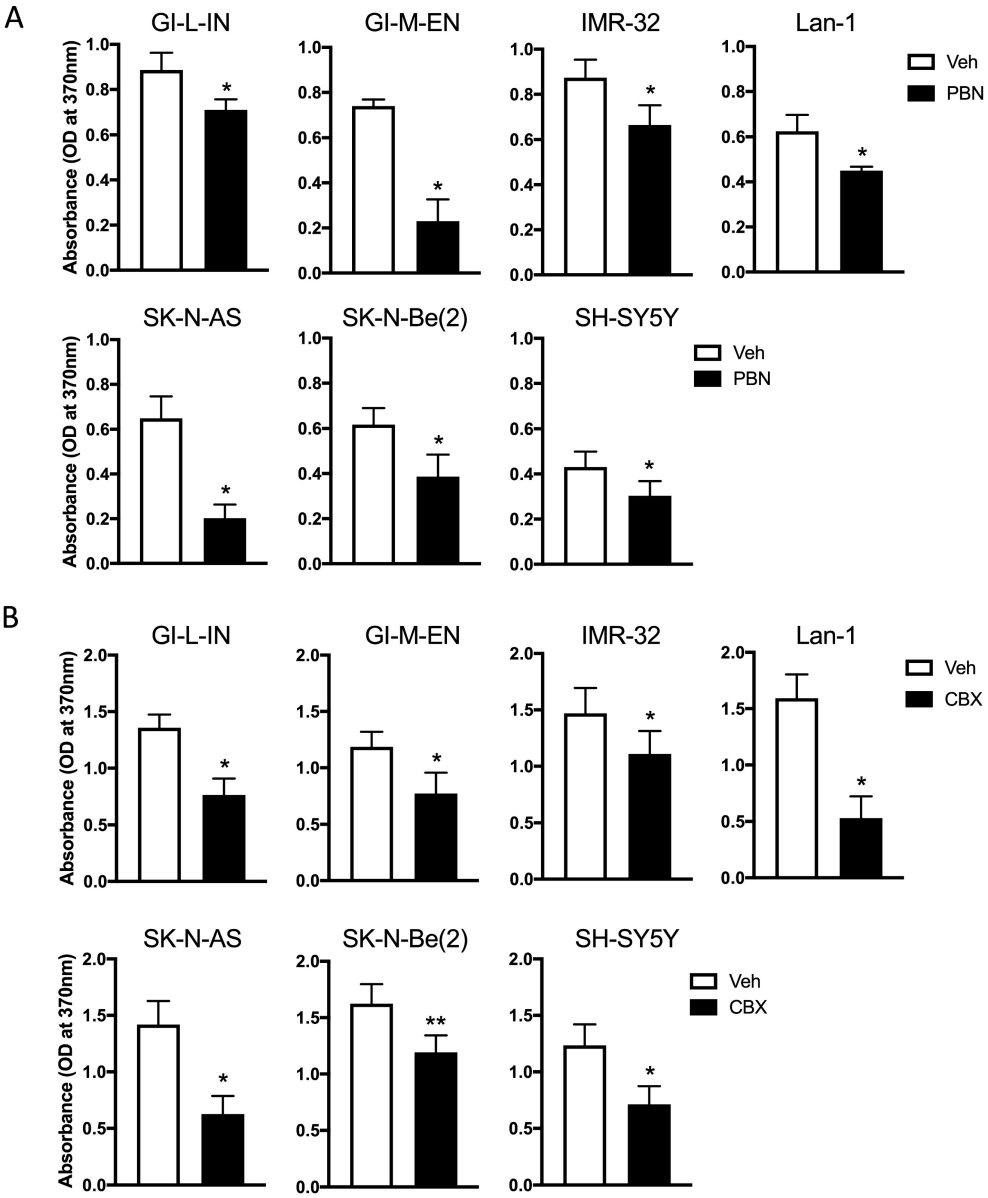
**Treatment with PBN or CBX inhibits NB cell proliferation.** BrdU incorporation (absorbance at 370 nm) was assessed after two days of treatment in our seven high-risk NB cell lines with **(A)** 1 mM PBN or **(B)** 100 μM CBX compared to cells treated with the vehicle control (Veh). Results are shown as mean ± SD. * p<0.05, ** p<0.01, compared to vehicle control (n≥3 independent biological replicates; paired two-tailed student's *t*-tests).

As treatment with PBN has been shown to inhibit the proliferation of the murine NB N2a cell line 9, our seven high-risk NB cell lines were treated with PBN over 48 hours prior to submitting them to a BrdU incorporation assay. As shown in Figure 3A, PBN treatment reduced the proliferation of all cell lines tested.

To determine whether CBX can also decrease NB cell proliferation, cells were treated with CBX for a period of 48 hours and then submitted to the BrdU incorporation assay (Fig. 3B). Our preliminary data using the same conditions as those used for testing the effect of PBN yielded absorbance values that were low for the assay detection range in some cell lines following CBX treatment. For this reason, the starting number of cells seeded for testing the effect of CBX on cell proliferation had to be increased compared to what was used to determine the effect of PBN. This is reflected in differences in the absorbance of the vehicle control cells in (Fig. 3A) compared to (Fig. 3B). Notably, treatment with CBX (Fig. 3B) significantly reduced the proliferation of all NB cell lines.

### Expression of the dominant-negative Y10F PANX1 mutant inhibits NB cell proliferation

**Figure 4:**
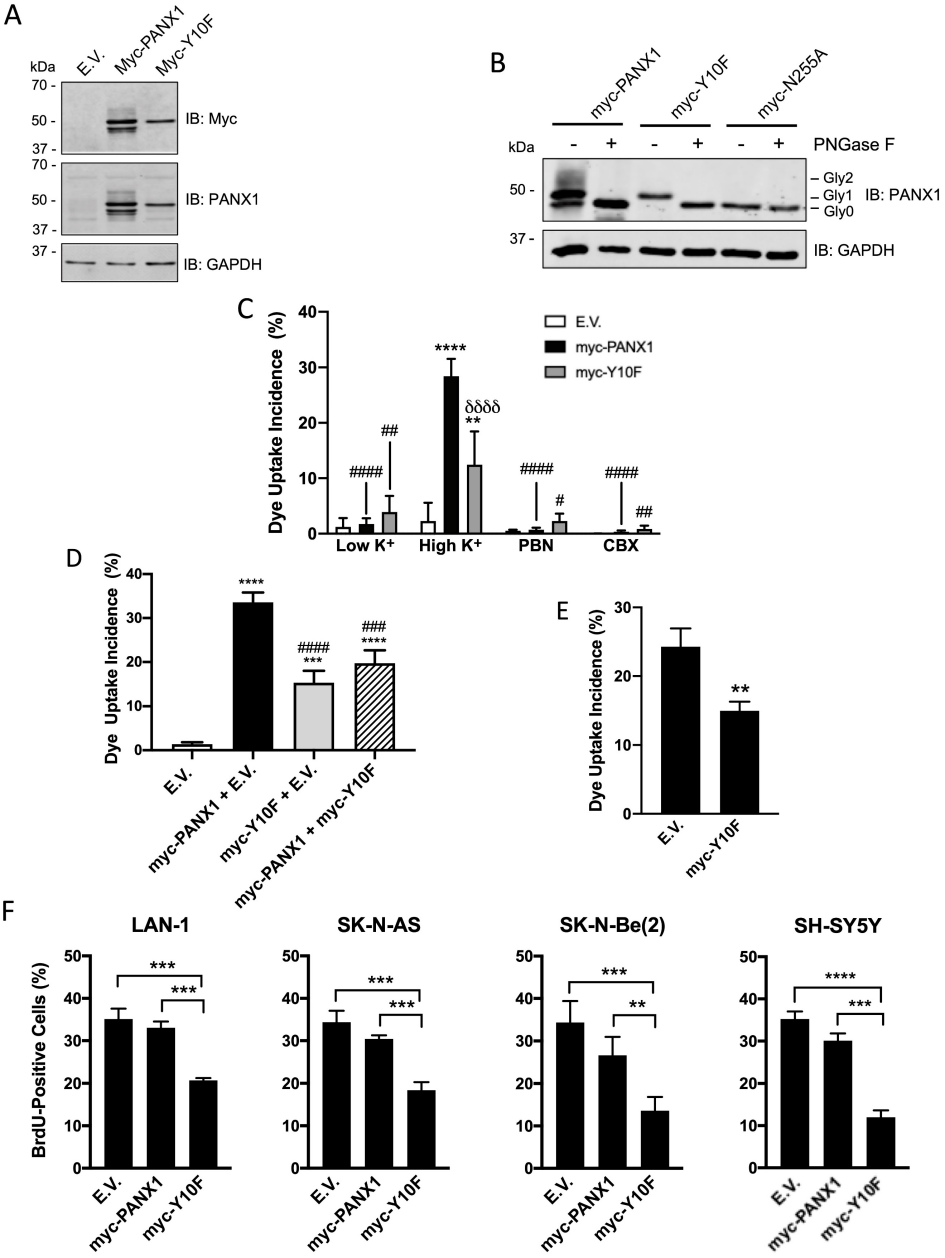
** Expression of the dominant-negative Y10F PANX1 mutant inhibits NB cell proliferation. (A)** Representative Western blot showing the myc-Y10F PANX1 mutant compared to its wild-type counterpart, the myc-PANX1, when expressed in HEK293T cells (n=3 independent biological replicates; 25 µg per well). GAPDH was used as a loading control. E.V.: Cells expressing the empty vector. **(B)** Representative Western blot showing myc-PANX1, myc-Y10F, and myc-N255A PANX1 following treatment with PNGase F (n=3 independent biological replicates; 50 µg per well). The various PANX1 species (unglycosylated state (Gly0), high-mannose (Gly1), and complex glycoprotein species (Gly2)) are indicated. GAPDH was used as a loading control. **(C)** Dye uptake incidence when Ad293 cells expressing myc-PANX1, myc-Y10F, or the empty vector (E.V.) were incubated in low K^+^ or high K^+^ solution. Dye uptake incidence was also quantified in the presence of 1 mM PBN and 100 μM CBX in high K^+^ condition. Results are expressed as mean ± SD. **** *p*<0.0001 compared to cells expressing the empty vector (E.V.) in the same culture condition; ^#^ *p*<0.05; ^##^ *p*<0.01; ^####^ *p*<0.0001 compared to their counterpart in the high K^+^ condition; and ^δδδδ^ *p*<0.0001 compared to cells expressing myc-PANX1 in the same culture condition (n=3 independent biological replicates; one-way ANOVA followed by Tukey's *post hoc* tests). **(D)** Dye uptake incidence measured in high K^+^ solution using Ad293 cells co-transfected (1:1 ratio) with myc-PANX1 and the empty vector (E.V), myc-Y10F and E.V., myc-PANX1 and myc-Y10F, or E.V. alone. Results are expressed as mean ± SD. *** *p*<0.001 and **** *p*<0.0001 compared to E.V. alone; ^###^ *p*<0.001 and ^####^ *p*<0.0001 compared to myc-PANX1 + E.V. (n=3 independent biological replicates; one-way ANOVA followed by Tukey's *post hoc* tests). **(E)** Dye uptake incidence following mechanical stimulation in SK-N-AS cells expressing myc-Y10F or the empty vector (E.V.). Results are expressed as mean ± SD. ** *p*<0.01 compared to cells expressing E.V. (n=3 independent biological replicates; unpaired two-tailed student's *t*-tests). **(F)** Percentage of BrdU-positive cells amongst four NB cell lines expressing either the empty vector (E.V.), myc-PANX1, or myc-Y10F. Results are expressed as mean ± SD. ** *p*<0.01, *** *p*<0.001, and **** *p*<0.0001 (n=3-4 independent biological replicates; one-way ANOVA followed by Tukey's *post hoc* tests).

In order to gain insight into the role of PANX1 in regulating NB cell proliferation, we targeted the PANX1 phosphorylation site at tyrosine (Y) residue 10 that was unveiled in a phosphoproteomic study screen of human NB cell lines 31. To this end, a myc-tagged PANX1 mutant (myc-Y10F (myc tag at the C-terminal end)) resistant to phosphorylation at residue 10 was generated by substituting the Y for a phenylalanine (F). As stated earlier, PANX1 is detected as multiple species by Western blot due to PTMs such as N-glycosylation 20. Myc-PANX1 (tagged at its C-terminus) was detected as 3 main species by Western blotting when expressed in HEK293T cells (Fig. 4A). On the other hand, the myc-Y10F mutant was detected as a single species recognized by both anti-PANX1 and anti-myc antibodies (Fig. 4A). Based on their sensitivity to treatment with deglycosylation enzymes, three PANX1 *N*-glycosylation species have been previously identified: Gly0 (unglycosylated), Gly1 (addition of high-mannose), and Gly2 (complex glycosylation) species 20. Murine Panx1 is known to be N-glycosylated at N254 22, which corresponds to residue N255 in human PANX1. To determine whether the myc-Y10F mutant is *N*-glycosylated, lysates were treated with Peptide-*N*-Glycosydase F (PNGase F) to remove *N*-linked oligosaccharides from glycoproteins (Fig. 4B). As a positive control, we used myc-PANX1 which migrated further as a single species (Gly0) after treatment with PNGase F, but not the negative control myc-N255A (myc tag at the C-terminus). Myc-Y10F migrated further after deglycosylation treatment and was then detected at the same molecular weight than myc-N255A, indicating that the myc-Y10F PANX1 mutant is expressed as a high-mannose Gly1 species.

To examine whether the channel activity of the myc-Y10F mutant is altered, this construct, as well as myc-PANX1 and the empty vector, were expressed in Ad293 cells. Even though these cells, which are derivative of human embryonic 293 cells, have improved adherence they still detach following mechanical stimulation. For this reason, a sulforhodamine B dye uptake assay in which PANX1 channels are activated by high extracellular K^+^ concentration 50 was used. When expressing the empty vector (E.V.), the cells showed similar dye uptake levels when incubated in high and low K^+^ (Fig. 4C). Dye uptake in high K^+^ was significantly increased when cells expressed myc-PANX1 or myc-Y10F (Fig. 4C), which were both reduced by PBN or CBX indicating the uptake measured was due to PANX1 channel activity (Fig. 4C). Notably, dye uptake incidence in cells expressing myc-Y10F was significantly lower when compared to that of myc-PANX1 (Fig. 4C), indicating that the Y10F mutant displays decreased channel activity. To assess whether the myc-Y10F mutant could have a dominant-negative effect, the myc-Y10F construct was co-transfected with wild-type PANX1 in Ad293 cells prior to performing the dye uptake assay in high K^+^ condition. As shown in Fig. 4D, cells co-transfected with myc-Y10F and myc-PANX1 showed significantly reduced dye uptake incidence compared to that of cells expressing myc-PANX1 together with the empty vector (E.V.) indicating that the myc-Y10F mutant acts in a dominant-negative manner. The myc-Y10F construct was also over-expressed in SK-N-AS cells, which express endogenous PANX1, prior to performing dye uptake assays. In accordance with the data obtained in Ad293 cells showing that the myc-Y10F mutant inhibits the channel function of wild-type PANX1, the expression of the myc-Y10F PANX1 mutant significantly reduced the dye uptake of SK-N-AS cells compared to that of cells expressing the empty vector (Fig. 4E).

Since the myc-Y10F PANX1 mutant has reduced channel activity compared to that of myc-PANX1 and inhibits PANX1 channel activity in a dominant-negative manner, we then wanted to determine if its expression could inhibit NB cell proliferation like what we have observed with PBN- or CBX-treated NB cell lines. To this end, the myc-Y10F and myc-PANX1 constructs, as well as the empty vector, were transfected in the four NB cell lines that showed the highest transfection efficiency in our hands, namely Lan-1, SK-N-AS, SK-N-Be(2), and SH-SY5Y. In all four NB cell lines, expression of myc-Y10F significantly suppressed cell proliferation in comparison to cells expressing the empty vector (E.V.) or myc-PANX1 (Fig. 4F). These results show that mutation of PANX1 tyrosine 10 inhibits its channel activity and NB cell proliferation. These findings further support the notion that inhibition of PANX1 channel activity reduces NB growth and may thus be therapeutically beneficial for this malignancy.

### Treatment with PBN or CBX inhibits NB 3D spheroid growth and induces regression of established spheroids

**Figure 5:**
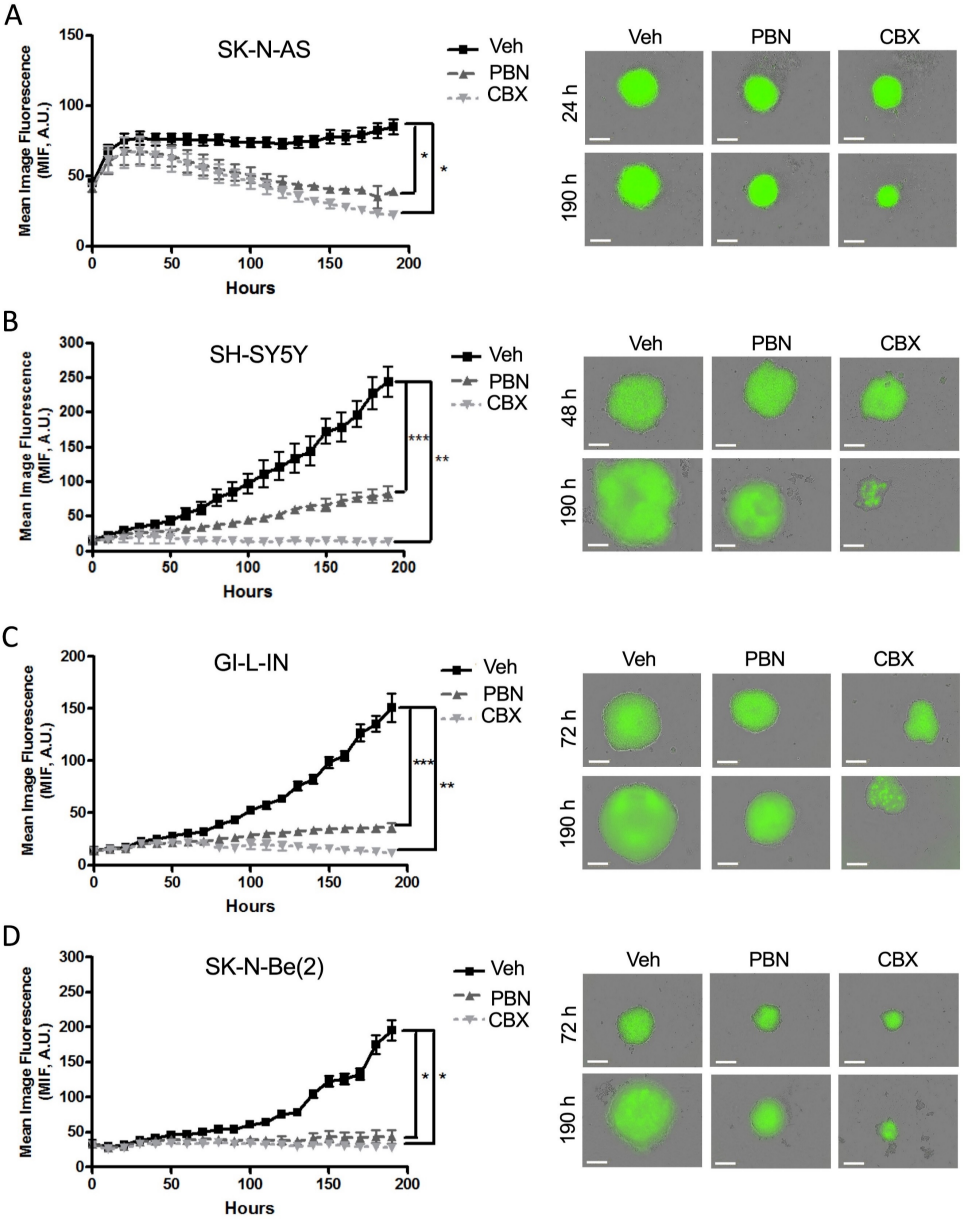
**Treatment with PBN or CBX inhibits 3D high-risk NB spheroid growth.** The NB cell lines **(A)** SK-N-AS, **(B)** SH-SY5Y, **(C)** GI-L-IN, and **(D)** SK-N-Be(2) were treated with 1 mM PBN, 100 μM CBX, or their vehicle control (Veh) at the time of seeding and treatment was continued over a period of 190 hours. Representative images taken at 24-72 hours and at 190 hours are shown. Mean image fluorescence (MIF), a measurement of spheroid size, was measured over time for all NB cell lines. Results are shown as mean image fluorescence (MIF) ± SD. * *p*<0.05, ** *p*<0.01, *** p<0.001 compared to treatment with the vehicle control (n=3 independent biological replicates; two-tailed student's *t*-tests at endpoint). A.U.: arbitrary units. Bars = 300 µm.

To investigate the therapeutic potential of PBN and CBX, tumor spheroid assays have been chosen as the functional endpoint since 3D culture models are thought to best mimic *in vivo* tumor growth 54. As spheroid growth and regression assays can be monitored and quantified easily using GFP (green fluorescent protein) with the fully quantitative real-time Incucyte^TM^ Imaging System 18, we have engineered four of our NB (SK-N-AS, SH-SY5Y, GI-L-IN, and SK-N-Be(2)) cell lines to stably express GFP. Aggregation and compaction of control SK-N-AS cells were first observed 24 hours after seeding on ultralow adherence plates (Fig. 5A), while SH-SY5Y spheroids had formed after 48 hours (Fig. 5B), with GI-L-IN and SK-N-Be(2) spheroids forming after 72 hours (Fig. 5C-D). While the growth of control spheroids continued to increase over time, treatment with PBN or CBX halted SH-SY5Y (Fig. 5B), GI-L-IN (Fig. 5C), and SK-N-Be(2) (Fig. 5D) spheroid growth. Notably, treatment with PBN or CBX caused spheroids formed by SK-N-AS cells to progressively diminish in size over time (Fig. 5A).

**Figure 6:**
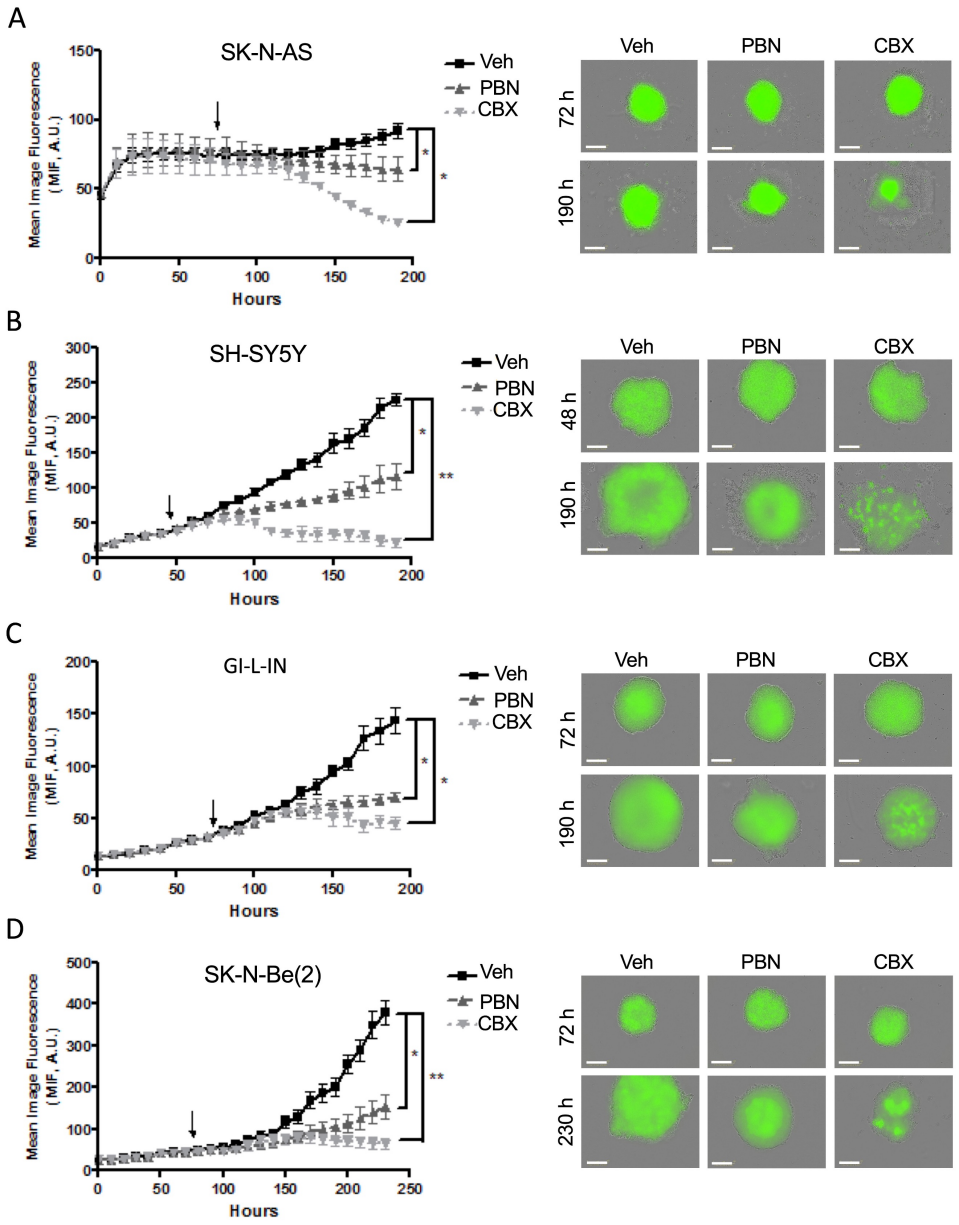
**Treatment with PBN or CBX induces regression of 3D high-risk NB spheroids.** In the spheroid regression assay, SK-N-AS **(A)**, SH-SY5Y **(B)**, GI-L-IN **(C)**, and SK-N-Be(2) **(D)** cells were first allowed to form established spheroids (48 hours for SH-SY5Y; and 72 hours for SK-N-AS, GI-L-IN, and SK-N-Be(2) - indicated by the arrow on the graphs) before being treated with 1 mM PBN, 100 μM CBX, or their vehicle control (Veh). Spheroid size was monitored for a total of 190-230 hours depending on the cell line. Representative images taken at 48-72 hours and 190-230 hours are shown. Results are expressed as mean image fluorescence (MIF) ± SD. * *p*<0.05, **p<0.01 compared to vehicle control (n=3 independent biological replicates; two-tailed student's *t*-tests at endpoint). A.U. arbitrary units. Bars = 300 μm.

To examine whether PBN and/or CBX could trigger tumor regression *in vitro*, treatment was next initiated once the cells had formed sizable spheroids after being in suspension (48 hours for SH-SY5Y cells and 72 hours for SK-N-AS, GI-L-IN, and SK-N-Be(2)). While control spheroids displayed ongoing growth, spheroids treated with PBN grew significantly slower for all 4 high-risk NB cell lines (Fig. 6A-D). Notably, established GI-L-IN and SK-N-Be(2) spheroids treated with CBX decreased in size reaching levels similar to that of the initial spheroids (Fig. 6C, D), while CBX treatment of established SK-N-AS and SH-SY-5Y spheroids resulted in a decrease in levels to at or below the original cell suspension (Fig. 6A, B), indicating regression of established NB spheroids. Taken together, our data demonstrate that treatment with PBN prevents NB spheroid growth, while treatment with CBX can also trigger their regression.

### Treatment with PBN or CBX reduces high-risk NB tumor progression in vivo

**Figure 7:**
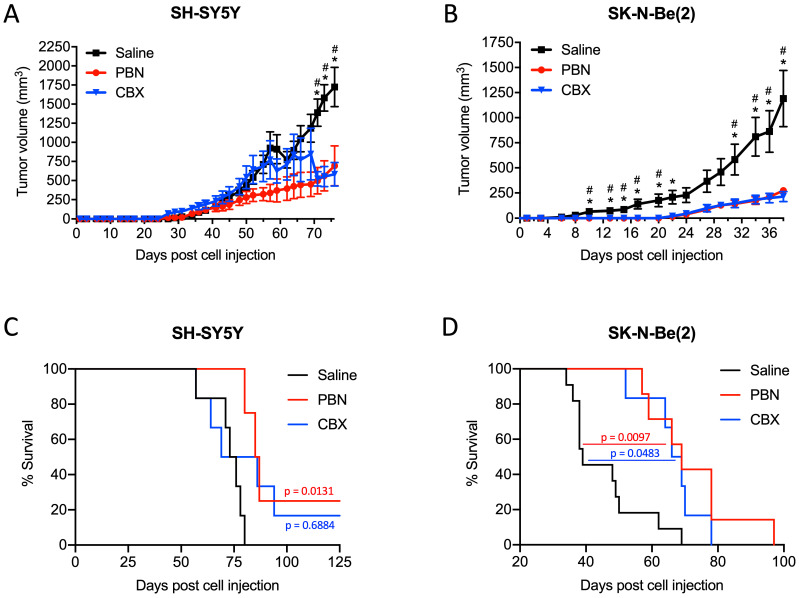
** PBN and CBX treatment reduces high-risk NB tumor progression *in vivo*.** SH-SY5Y and SK-N-Be(2) cells were injected subcutaneously in the flank of CD1 nu/nu mice (randomly assigned). PBN (250 mg/kg), CBX (30 mg/kg), or saline was injected daily. Tumor volumes **(A, B)** were measured until the saline control reached humane endpoint or the maximum tumor size allowed at our institution (~2,000 mm^3^). Day 0 denotes the day of cell injection. Results are expressed as mean ± SEM. * *p*<0.05 compared to PBN; # *p*<0.05 compared to CBX (n=6-13 mice per group; one-way ANOVA followed by Tukey's *post hoc* tests). Kaplan-Meier curves show survival of PBN-, CBX-, and saline-treated mice injected with SH-SY5Y **(C)** or SK-N-Be(2) **(D)** cells. Statistical significances were determined using the Gehan-Breslow-Wilcoxon test (this method gives more weight to deaths at early time points) and indicated in red (PBN compared to saline) and blue (CBX compared to saline) on the graphs.

Based on these results, we next wanted to validate the beneficial effect of PBN and CBX *in vivo*. To this end, SH-SY5Y and SK-N-Be(2) cells were injected into the flank of CD1 nu/nu mice and then treated with either saline, PBN (250 mg/kg body weight) 51, 52, or CBX (30 mg/kg body weight) 53 daily until tumors reached 1500-2000 mm^3^, at which time point mice were sacrificed. Treatment with PBN or CBX both significantly repressed the growth of tumors formed by SH-SY5Y (Fig. 7A) and SK-N-Be(2) cells (Fig. 7B). While not observed with SH-SY5Y cells, treatment with PBN or CBX also delayed SK-N-Be(2) tumor onset. Indeed, PBN- and CBX-treated mice did not present with any tumors until 22 days after injection of SK-N-Be(2) cells, while all saline-treated control mice had tumors 6 days post-injection. While the median survival was not statistically different between CBX and saline-treated mice bearing SH-SY5Y xenografts, the median survival was significantly increased in PBN-treated mice (Fig. 7C). As for mice bearing SK-N-Be(2) xenografts, median survival was significantly increased by both PBN and CBX treatment (Fig. 7D). Taken together, these *in vivo* data demonstrate a therapeutic benefit of using PBN or CBX to reduce high-risk NB progression.

## Discussion

Our findings show that PANX1 is expressed in human NB tumors and that PANX1 levels are similar between the various NB stages. However, we found that high *PANX1* expression is associated with a reduced survival probability in high-risk NB patients. *In vitro*, our findings indicated that PANX1 is expressed and forms active channels in all seven high-risk NB cell lines assessed. Interestingly, a higher molecular weight PANX1 species (~50 kDa) was detected by Western blotting in NB cell lines, but not in neurons, likely reflecting different post-translational modifications 20. We have previously shown that the endogenous ~50 kDa PANX1 species expressed in skeletal muscle cells corresponds to a glycosylated form of PANX1 as, after deglycosylation treatment, it migrated further at ~38 kDa 10. We have also previously demonstrated that the levels of this higher molecular weight species (~50 kDa) of PANX1 was decreased in the fully differentiated adult skeletal muscle tissue, compared to that of partially differentiated fetal tissue, whereas the lower molecular weight forms of PANX1 were more abundant 10. Accordingly, the ~50 kDa PANX1 species expressed in the murine neuroblastoma cell line N2a was decreased during their differentiation in culture 13. The variation in the PANX1 species seen in our panel of human high-risk NB cell lines compared to that of neurons may thus reflect that these NB cells are poorly differentiated, i.e. features of neuronal differentiation are low or absent.

The potential therapeutic benefit of using pharmacological inhibition of PANX1 channels by PBN or CBX has been previously reported in the context of human breast cancer *in vivo* 17, 32, human melanoma cells *in vitro* and in chicken embryo xenografts 35, mouse testicular cancer cells *in vitro* 55, and a mouse neuroblastoma cell line *in vitro* 9. As we are interested in the translational aspect of these drugs, we used four to seven high-risk NB cell lines, each with different genetic characteristics and clinical features, to demonstrate that treatment with PBN or CBX reduced NB tumor cell proliferation *in vitro*, halted 3D spheroid growth, and either arrested growth or induced regression of established 3D spheroids. Moreover, using two different high-risk NB cell lines, we have shown that treatment with PBN or CBX significantly reduced tumor progression *in vivo*.

The inhibition of NB growth by PBN and CBX indicate a role for PANX1 channel function in regulating this process. It has been previously shown that Panx1 increases the proliferation of the murine NB-derived cell line N2a through the release of ATP, which subsequently activates the P2 purinergic receptors 9. Blocking P2 receptors using pyridoxalphosphate-6-azophenyl-2',4'-disulfonic acid (PPADS) also reduced N2a cell proliferation 9. It is thus tempting to speculate that ATP release by PANX1 and subsequent P2 receptors activation are also involved in the regulation of human NB growth. A feedback mechanism may also exist in which ATP activates PANX1 channels in a P2-dependent manner 56. While the molecular mechanism by which PANX1 channels regulate NB malignant properties remain to be investigated and the knowledge of the signaling pathways triggered by PANX1 in cancer remain to be further elucidated, some studies have revealed a link between PANX1 and the epithelial-to-mesenchymal transition (EMT). In most cases, benign tumor cells acquire metastatic properties during tumor progression due to EMT. In breast cancer cells, in which PANX1 over-expression is correlated with poorer overall survival, the expression of genes involved in the EMT pathway correlated positively with PANX1 expression 17. Moreover, PANX1 channel inhibition or gene ablation down-regulated EMT pathway genes and reduced the metastatic potential of breast cancer cells 17. PANX1/Panx1 promoted EMT in hepatocellular carcinoma cell lines via phosphorylation of AKT 34. Blocking PANX1 channels reduced ATP release, as well as the growth and tumorigenic properties of human melanoma cells 35. Moreover, β-catenin levels were decreased upon PANX1 silencing unveiling a potential role for PANX1 in regulating signaling through the Wnt (wingless-type MMTV integration site family)-β-catenin pathway in human melanoma 35. Loss of distinct Wnt pathway components has the potency to alter the behavior of tumor cells and their environment 57. As NB and melanoma are both neural crest-derived tumors that may have a shared genetic basis 58, future studies will investigate the role of EMT and the Wnt-β-catenin pathway, in addition to that of ATP release and P2 receptors, in the PANX1-mediated regulation of NB malignant properties.

Strengthening the findings that PANX1 channel inhibition has therapeutic potential for high-risk NB, mutation of the PANX1 Y10 residue inhibited PANX1 channel activity and NB cell proliferation. While several PTMs have been shown to regulate channel activity and function 20, this is the first study to suggest a potential role for PANX1 phosphorylation on tyrosine 10. It has been shown that PANX1-mediated ATP release and vasoconstriction involves constitutive phosphorylation of PANX1 tyr-198 by Src 27. This phosphorylation may regulate PANX1 channel activity as a motif containing the residue Y198 has been implicated in the activation of PANX1 channels 59. More recently, a mutated form of PANX1, Y150F, has been found in a melanoma patient tumor 28. This mutation prevented phosphorylation at Y150 as well as N-glycosylation of PANX1 and increased its intracellular localization 28. As we have shown that the Y10 mutation affects PANX1 glycosylation, it is possible that the inhibitory effect of the Y10F mutation on PANX1 channel activity is consequent to the reduction of complex glycosylation instead of a direct correlation with its Y10 phosphorylation status. Mutation of Y10 may also prevent a key interaction of PANX1 with another protein, leading to an inhibitory effect on channel function and subsequently on cell proliferation.

Like PANX1 phosphorylation on Y198, the phosphorylation of PANX1 on tyrosine residues 150 and 308 may also be Src-dependent 28, 30. While the identity of the kinase and the mechanism of phosphorylation at PANX1 Y10 are currently unknown, one potential candidate is Src due to its over-expression in NB 60, 61. As PANX1 is expressed throughout the body, future work should be aimed at identifying the kinase responsible for PANX1 phosphorylation on Y10. This may allow the generation of a specific PANX1 inhibition strategy in NB as it has been previously done using interfering peptides in other disease contexts. For example, blocking the phosphorylation and activation of PANX1 by Src during ischemia through administration of the interfering peptide TAT-PANX_308_ before or 2h after stroke onset reduced lesion size and sensorimotor deficits in adult rats 62.

Together our data indicate that inhibition of PANX1 channels, either via treatment with PBN or CBX, or by mutating PANX1 on tyrosine 10, significantly reduces the malignant properties of human high-risk NB. Our findings also suggest that the repurposing of PBN and CBX may provide a new therapeutic approach for this patient population.
